# Environmental Durability of Bio-Based and Synthetic Thermoplastic Composites in Large-Format Additive Manufacturing

**DOI:** 10.3390/polym16060787

**Published:** 2024-03-12

**Authors:** Felipe A. Saavedra-Rojas, Sunil Bhandari, Roberto A. Lopez-Anido

**Affiliations:** 1 Advanced Structures and Composite Center, University of Maine, Orono, ME 04469, USA; felipe.saavedra@maine.edu (F.A.S.-R.); rla@maine.edu (R.A.L.-A.); 2 Department of Civil and Environmental Engineering, University of Maine, Orono, ME 04469, USA

**Keywords:** large-format additive manufacturing, 3D printing, durability, accelerated testing, moisture, freeze–thaw, composite, bio-based, carbon fiber

## Abstract

This research investigates the durability of large-format 3D-printed thermoplastic composite material systems under environmental exposure conditions of moisture and freeze–thaw. Durability was evaluated for two bio-based composite material systems, namely wood-fiber-reinforced semi-crystalline polylactic acid (WF/PLA) and wood-fiber-reinforced amorphous polylactic acid (WF/aPLA), and one conventionally used synthetic material system, namely short-carbon-fiber-reinforced acrylonitrile butadiene styrene (CF/ABS). The moisture absorption, coefficient of moisture expansion, and reduction of relevant mechanical properties—flexural strength and flexural modulus—after accelerated exposure were experimentally characterized. The results showed that the large-format 3D-printed parts made from bio-based thermoplastic polymer composites, compared to conventional polymer composites, were more susceptible to moisture and freeze–thaw exposure, with higher moisture absorption and greater reductions in mechanical properties.

## 1. Introduction

In recent years, the use of additive manufacturing (AM) to make functional products has increased significantly. The expansion of 3D printing technology to include larger-scale capabilities has the potential to revolutionize various industries by offering more efficient, cost-effective, and flexible manufacturing solutions [1]. Bishop et al. [2] highlighted the usage of large-format additive manufacturing for rapid manufacturing processes in response to emergency shortages. Roschli et al. [3] demonstrated the use of precast concrete molds fabricated for casting architectural building components using additive manufacturing. Bhandari et al. presented the use of 3D-printed formworks for a precast concrete pier cap of a highway bridge [4], formworks for window openings for a precast concrete parking garage wall system [5], and formworks for casting precast concrete ballast retainers for railroad bridges [6]. Peters [7] studied the potential and advantages of flexible formwork for precast concrete architectural applications, showing that it can be implemented immediately in the construction industry since it improves an already known technique and material.

Large-format AM has also been used to manufacture polymer composite structures that are expected to last for long timespans. Bhandari et al. [8] demonstrated the use of 3D-printed diffusers for the rehabilitation of highway culverts. The University of Maine and Oak Ridge National Laboratory collaborated to design and manufacture a bio-based additively manufactured house prototype known as BioHome3D [9]. Liedtka [10] evaluated the life cycle, embodied energy, and sustainability potential for large-format additive manufacturing of 3D-printed modular houses. Conventional polymer composite systems derived from petroleum products as well as novel bio-based polymer composite systems are being used as feedstock material in large-format additive manufacturing. Van de Werken et al. [11] reviewed the use and effectiveness of short carbon fiber reinforcement in different polymer composite systems, used as a feedstock material in large-format additive manufacturing. Zhao et al. [12] used poplar-biofiber-reinforced polylactic acid (PLA) to manufacture podium supports and found that the porous and hollow microstructures in poplar fibers enabled better interfacial bonding with the PLA polymer. Lamm et al. [13] reviewed the use of natural fillers in the polymer composite systems used in large-format additive manufacturing.

The increasing adoption of large-format AM for the manufacturing of long-standing structures with outdoor exposure has necessitated the development of a better understanding of the mechanical durability of large-format extrusion-based AM-produced polymer composite parts [14]. Specifically, the durability of a material or structure is summarized as “its ability to resist cracking, oxidation, chemical degradation, delamination, wear, and/or the effects of foreign object damage for a specified period, under the appropriate load conditions, under specified environmental conditions” [15]. The durability of various conventionally manufactured synthetic as well as bio-based polymer composites has been well studied. The durability of polymer composites is divided into two broad categories: structural durability and aesthetic durability [16]. Both categories are important since, from the structural durability point of view, polymer composites need to withstand service loads while, at the same time, maintaining the required aesthetics during their service life. From the structural point of view, durability can be defined as the ability of a building structure to remain fit for the design purpose during its service life [17]. Accelerated testing uses several test techniques to cut the lifespan of products or speed up the performance degradation of such products [18]. Stamboulis et al. [19] studied the durability of compression-molded flax-fiber-reinforced polypropylene composites. Stark et al. [20] studied the outdoor durability of wood polymer composites (WPC) made of high-density polyethylene (HDPE) with 50% wood flour, comparing extruded, compression-molded, and injection-molded WPC. For moisture, the hydroxyl groups on wood or other lignocellulosic materials are primarily responsible for water absorption. As the wood particle absorbs moisture, it swells, producing stresses in the WPC matrix and creating microcracks. Swelling also creates stress in the wood particles. Once the material is dried, there is no adhesion at the matrix and wood particle interface, creating voids that water will penetrate during later exposure, affecting its durability. In a ten-year field study, Gardner et al. [21] inserted stakes made of polypropylene (PP) with 49–52% wood flour in the ground to evaluate decay, termite ratings, surface weathering, biological colonization, and dimensional changes. In addition, flexural tests were conducted to evaluate the flexural properties after ten years, showing a decrease or no change depending on the composite formulation. Malpot et al. [22] investigated the effect of moisture ingress on the fatigue behavior of glass-fiber-reinforced polyamide manufactured using resin transfer molding. The study found that moisture ingress significantly increased the fiber fracture for fatigue loadings with medium to high stress ratios. Pomies and Carlsson [23] studied the effect of moisture on injection-molded glass-fiber-reinforced polyphenylene sulfide and found degradation in the fiber–matrix interface due to moisture ingress.

Researchers have studied the environmental durability of desktop-scale 3D-printed thermoplastic composites. Celestine et al. [24] studied the mechanical moisture-ingress-induced degradation of 3D-printed and injection-molded nylon polymer parts. The study found that the rate of absorption for the 3D-printed specimens was higher than that of the injection-molded specimens. Cormier and Poddar [25] highlighted that printing voids, the interlayer bond strength, and the fiber strength affect the durability of parts fabricated via additive manufacturing. These properties are influenced by the processing settings and material selection. The material structure in large-format polymer-extrusion-based 3D parts makes the material more susceptible to moisture absorption and water ingress, which affects the durability. Banjo et al. [26] reported a high water absorption rate for desktop-scale 3D-printed nylon and PLA and corresponding physical and mechanical property degradation, when immersed in water at high temperatures of 70 °C. However, the loss in mechanical properties was negligible when the immersion was at a temperature of 20 °C. Kaknuru and Pochiraju [27] studied the uptake of moisture in desktop-scale 3D-printed ABS and PLA polymers and highlighted the effects of moisture penetration on the mechanical properties of the 3D-printed parts. Xiao et al. [28] studied the degradation in the mechanical properties of 3D-printed polyethylene due to exposure to ultraviolet radiation and found that such exposure caused a reduction in tensile strength, modulus, ductility, and toughness. Dizon et al. [29] studied the effect of post-processing on 3D-printed polymer composite parts and found that post-processing could be used to improve the durability of such parts. Afshar and Mihut [30] found that the durability of 3D-printed polymer composite parts could be improved by depositing a thin metallic copper film on the surface of the 3D-printed parts. Glowacki et al. [31] studied the effect of freeze–thaw cycling on the durability of 3D-printed acrylonitrile butadiene styrene and polylactic acid and found that one cycle lasting seven days was enough to alter the mechanical properties of the materials.

Although studies have been carried out to investigate the durability of thermoplastic polymer composites manufactured using desktop-scale 3D printing, the durability of thermoplastic composite materials manufactured with large-format 3D printing needs further investigation. Grassi et al. [32] studied the durability of wood-fiber-reinforced polylactic acid and polypropylene for desert climates. The study focused on the durability of large-format 3D-printed structures against thermal and ultraviolet exposure. Large-scale 3D-printed parts for exterior use are exposed to freeze–thaw cycles and moisture. This exposure affects the durability of the part in service. The voids and imperfect fusion between layers and the micro-porosity within the beads are more pronounced in parts manufactured with large-format additive manufacturing [33]. Such a material structure in large-format polymer-extrusion-based 3D parts makes the material more susceptible to moisture absorption and water ingress, which affects the durability. Hence, a durability study of large-format 3D-printed polymer composite materials is necessary to evaluate their use and guide the design of long-standing structures.

The objectives of this study are to

1. Investigate the moisture absorption behavior of bio-based and synthetic polymer composites manufactured using large-format additive manufacturing;
2. Evaluate the dimensional changes of bio-based 3D-printed polymer composites caused by moisture ingress;
3. Characterize the mechanical property degradation of large-format 3D-printed bio-based and synthetic polymer composites due to moisture ingress; and
4. Characterize the mechanical property degradation of large-format 3D-printed bio-based and synthetic polymer composites due to freeze–thaw cycling.

## 2. Materials and Methods

### 2.1. Materials and Sample Preparation

#### 2.1.1. Materials

This study used two bio-based polymer composites and a synthetic polymer composite.

- WF/PLA, or wood fiber (WF)-reinforced polylactic acid (PLA), is a bio-based polymer composite with a conventional semi-crystalline PLA polymer. HiFill PLA 1816 3DP with 20% fiber reinforcement by weight, supplied by Techmer PM, Clinton, TN, USA, was used as the feedstock material for large-format 3D printing.
- WF/aPLA, or WF-reinforced amorphous PLA, is a bio-based polymer composite with a novel amorphous PLA polymer. PLA 3120 WF PEL with 20% fiber reinforcement by weight, supplied by Jabil, Chaska, MN, USA, was used as the feedstock material for large-format 3D printing.
- CF/ABS, or short-carbon-fiber-reinforced acrylonitrile butadiene styrene (ABS), is a synthetic material system with a petroleum-derived ABS polymer. Electrafil ABS 1501 3DP with 20% fiber reinforcement by weight, supplied by Techmer PM, Clinton, TN, USA, was used as the feedstock material for large-format 3D printing.

**Figure 1:**
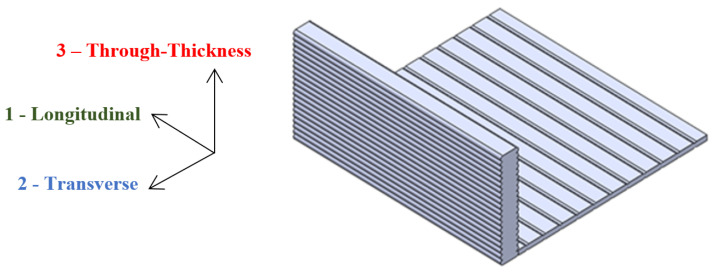
Material direction convention adopted for large-format 3D-printed specimens.

The 3D-printed parts exhibited orthotropic mechanical behavior [34,35]. Figure 1 shows the principal material direction convention adopted for the large-format 3D-printed specimens. Direction 1 is along the bead, direction 2 is perpendicular to the bead in the same plane of deposition, and direction 3 is normal to the plane of deposition.

#### 2.1.2. Specimen Preparation

**Figure 2:**
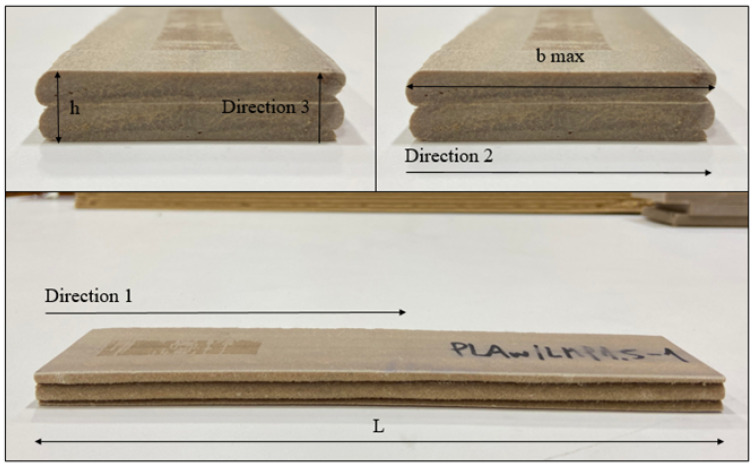
The 3D-printed specimens with dimensions and material direction.

The 3D-printed panels were manufactured using large-format 3D printing using the Ingersoll Masterprint 3D printing equipment. A bead width of 19.1 mm (0.75 inches) and a bead height of 5.08 mm (0.20 inch) were used for the manufacturing of the specimens. The extrusion temperature at the nozzle for CF/ABS, WF/PLA, and WF/aPLA was 240 °C, 207 °C, and 210 °C, respectively. Figure 2 shows the principal material directions in the specimen. The specimens were 196 mm long, 38.2 mm wide, and 10.2 mm thick. The specimens were two beads thick and two beads wide. The specimens consisted of at least one interlayer interface and one intralayer interface, so that the effect of the interface on water ingress, hygroscopic expansion, and mechanical properties was taken into account.

### 2.2. Experimental Methods

Three sets of 3D-printed specimens were prepared. Each set consisted of three different materials. The first set of 3D-printed specimens served as the baseline specimens. These baseline specimens were used to characterize the as-printed mechanical properties. The second set of 3D-printed specimens was subjected to accelerated water immersion. The third set of specimens was subjected to freeze–thaw cycling.

#### 2.2.1. Accelerated Water Immersion

The ASTM D5229 [36] test standard was adopted to evaluate the moisture absorption and equilibrium conditioning of the 3D-printed polymer composite specimens. The selected temperature for oven drying and water absorption was 40 °C (below the glass transition temperature ($T_{g}$) of WF/PLA 60 °C, being the lowest $T_{g}$ of the three materials). Specimens were oven-dried at 40 °C for 24 h. Specimens were immersed in distilled water at 40 °C after oven drying, and the conditioning time was 81 days. Moisture content was calculated according to the ASTM D5229 standard. Sorption curves were generated according to the ASTM D5229 standard. The length was measured using a calibrated steel ruler and the width and height were measured using calibrated vernier calipers. The coefficient of moisture expansion (CME) β for the specimens was calculated using Equations (1) and (2) [37,38].

(1) $$\beta =\frac{\varepsilon_{H}}{M(\%)}$$

(2) $$\varepsilon =\frac{\Delta l}{l_{0}}$$

where

$\varepsilon_{H}$ = moisture strain;
Δl = change in dimensions of the specimen;
$l_{0}$ = initial dimensions;
β = coefficient of moisture expansion for the composite material;
M(%) = specimen moisture content as a percentage.

#### 2.2.2. Freeze–Thaw Cycling

Freeze–thaw cycling was conducted according to ASTM D7992 [39]. A minimum of three specimens were subjected to the freeze–thaw cycles below to determine the effect of freeze–thaw exposure. Test specimens were submerged underwater for 24 h. The specimens were then placed in a freezer at −29 °C (−20 °F) for 24 h. After being frozen, the specimens were returned to room temperature for 24 h. This process comprised one hygrothermal cycle. The procedure was repeated two more times for a total of three cycles of water submersion, freezing, and thawing. After three freeze–thaw cycles, the specimens were allowed to return to room temperature, followed by flexure testing.

### 2.3. Evaluation of Mechanical Properties Using Flexure Tests

**Figure 3:**
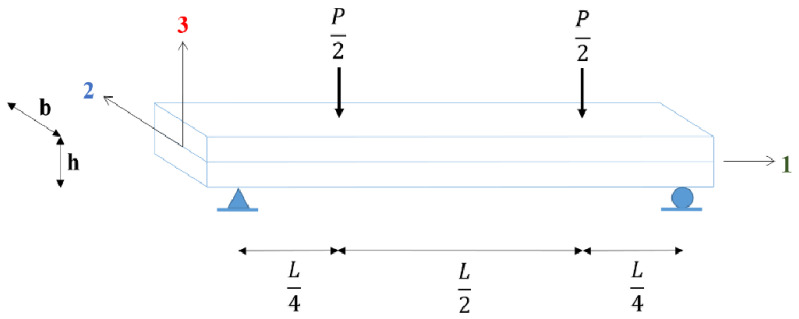
Beam specimen loading configuration and material directions.

Procedure B of the ASTM D7264 standard test method [40] was adopted to evaluate the flexural modulus and flexural strength of the specimens. A beam specimen with a rectangular cross-section was simply supported and loaded in four-point bending. A constant displacement rate of 4.34 mm/min was adopted. Figure 3 illustrates the loading configuration and the principal material axes of the beam specimen. The specimen was loaded in flexure along the direction of the deposited beads (direction—1). The length of the specimen was 196 mm, the width was 38.2 mm, and the height was 10.2 mm. The specimen dimensions were selected based on the bead geometry of the 3D-printed parts, with the width of the flexure test specimen equal to the width of two extruded beads and the specimen height equal to the thickness of two extruded beads. The loaded span was 180 mm with an 8 mm overhang on both sides. The loads were applied at a distance of 90 mm from the supports.

**Figure 4:**
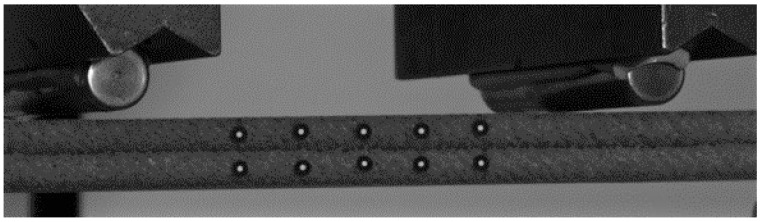
Central span of specimen loaded in four-point bending with single-point markers.

An ARAMIS (v 6.3) 3D-Digital Image Correlation (DIC) system from GOM GmbH, Braunschweig, Germany was used to record the strains on the test specimens’ surfaces during testing. The specimens were prepared for the DIC measurement system by placing uncoded white single-point markers of 0.8 mm diameter. Figure 4 shows the single-point markers used for DIC to evaluate the displacements at different locations in the specimens during testing. The displacements were used by the DIC software to calculate the axial strains in the 3D-printed specimens. The flexure tests were performed on a 100 kN servo-hydraulic load frame from Instron, Norwood, MA, equipped with side-loading hydraulic grips, at a temperature of 23 ± 2 °C and 50 ± 5% relative humidity in an environmentally controlled test lab at the Advanced Structures and Composites Center at the University of Maine in Orono, Maine.

Flexural strength was calculated using Equation (3).

(3) $$\sigma_{b}=\frac{3PL}{4bh^{2}}$$

where

$\sigma_{b}$ = flexural strength;
*P* = total applied load;
*L* = beam span;
*b* = beam width;
*h* = beam height.

## 3. Results and Discussion

### 3.1. Water Immersion Test Results

Moisture diffusion occurred primarily through the surfaces normal to directions 2 and 3 in the specimen. Directions 2 and 3 in relation to the 3D-printed specimens are shown in Figure 2. The average moisture content measured for WF/PLA, WF/aPLA, and CF/ABS specimens at the end of accelerated moisture exposure are shown in Table 1.

After water immersion for 81 days, WF/aPLA was the material with the highest moisture content of 9.23%, followed by WF/PLA with moisture content of 7.74%, and finally CF/ABS with moisture content of 1.35%. Similar results were observed for WPC- and CF-reinforced polymer composites by other researchers. Wang et al. [41] reported moisture of 10 % to 16 % in different wood plastic composites after water immersion for 200 days. Kim et al. [42] observed moisture absorption of 12% to 16% for different wood polymer composites manufactured using polypropylene and different wood species after water immersion for 100 days. Huang et al. [43] observed moisture absorption of 5% to 6% for different wood polymer composites subjected to water immersion for 40 days.

**Figure 5:**
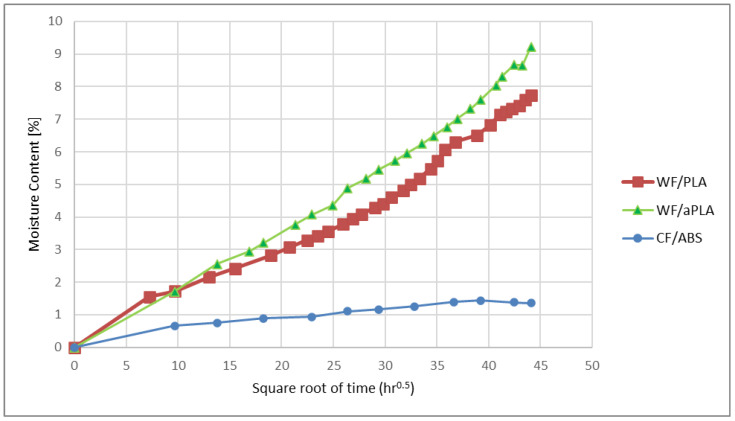
Sorption curves for WF/PLA, WF/aPLA, and CF/ABS.

Sorption curves were generated to visualize the variation in moisture over time. Figure 5 illustrates the sorption curves for WF/PLA, WF/aPLA, and CF/ABS specimens. The bio-based composites gained moisture more rapidly compared to the synthetic polymer composite. WF/aPLA showed the highest rate of increase in moisture with time, followed by WF/PLA. CF/ABS had the lowest rate of water uptake with time. The moisture content of the bio-based polymer composite specimens increased with time and did not reach equilibrium at the end of the testing period. For bio-based polymer composites, the rate of water absorption did not decrease with time. This suggests that mechanisms other than the simple sorption of water by the polymer and reinforcing fiber might have been in action. Jiang et al. [44] reported similar water uptake behavior in conventionally manufactured Jute/PLA composites, where the water uptake of such composites did not reach saturation levels. The research work showed that the water absorption proceeded in three different stages: water absorption by the polymer matrix and wood fiber, crack formation at the matrix–fiber interface, and further microcracking due to the hydrolysis of the matrix. Further study is necessary to identify the cause of this behavior observed in bio-based large-format 3D-printed polymers. The rate of water uptake with time decreased for CF/ABS polymers. The moisture content of CF/ABS specimens increased and approached saturation levels at the end of the testing period. Aniskevich et al. [45] reported similar moisture uptake behavior in 3D-printed ABS polymers.

The coefficients of moisture expansion of the specimens subjected to the accelerated water immersion tests were evaluated. The CF-ABS specimens did not exhibit any change in dimensions when the length was measured with a calibrated ruler and the width and height were measured with vernier calipers. Table 2 shows the coefficient of moisture expansion for WF/aPLA and WF/PLA specimens.

$\beta_{1}$, $\beta_{2}$, and $\beta_{3}$ are the coefficients of moisture expansion in directions 1, 2, and 3, respectively. The coefficients of moisture expansion showed high values of variation, especially for WF/PLA specimens. In order to compare the CMEs of the materials in different directions and check whether the specimens exhibited orthotropic moisture expansion behavior, an ANOVA test was performed. A *p*-value of less than 0.05 was considered statistically significant. The results of the ANOVA test are presented in Table 3.

The results from the ANOVA test showed that

- The large-format 3D-printed WF/aPLA exhibited orthotropic moisture expansion behavior, i.e., the CME in each of the three principal material directions was unique;
- The difference between $\beta_{1}$ and $\beta_{2}$ was not statistically significant; the large-format 3D-printed WF/PLA presented in-plane isotropy, with planes 1–2 as the planes of isotropy.

### 3.2. Flexure Test Results

#### Baseline Results

Table 4 presents the average flexural strength and flexural modulus of the baseline sets. The number of specimens and the coefficient of variation for strength and moduli calculation are listed in parentheses.

**Figure 6:**
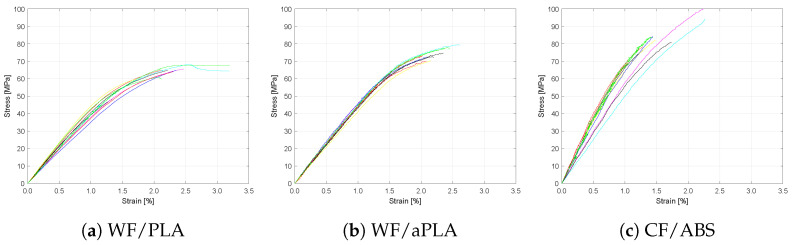
Flexural stress vs. strain curves for baseline specimens.

Figure 6 shows the flexural stress vs. strain test results for the baseline specimens manufactured using different materials. Different colored curves represent the stress-strain curves for individual specimens. The flexural strength of the 3D-printed CF/ABS was the highest among the three polymer composites, while that of the WF/PLA polymer composite was the lowest. The 3D-printed WF/PLA polymer composites were more ductile, with a strain to failure of 2.7% compared to that of 2.2% of WF/aPLA and 1.6% of CF/ABS. The CF-ABS specimens also showed higher variability in flexural strength and flexural modulus, with a COV of 11% in strength and 15% in flexural modulus.

### 3.3. Flexure Test Results of Specimens Subjected to Accelerated Water Immersion Tests

The WF/PLA specimens failed at a preload of 40 N, indicating severe degradation after 81 days of accelerated water immersion aging. Table 5 presents the average flexural strength and flexural modulus of the WF/aPLA and CF/ABS sets subjected to accelerated water immersion aging. A 73% reduction in flexural strength and a 49% reduction in flexural modulus was observed in WF/aPLA composite specimens. A 49% reduction in flexural strength and an 18% reduction in flexural modulus was observed for CF/ABS composite specimens.

**Figure 7:**
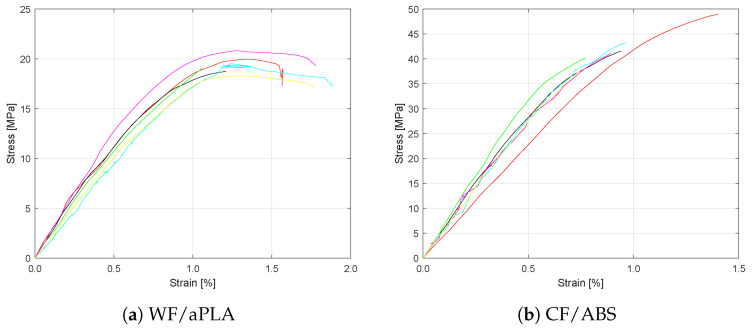
Flexural stress vs. strain curves for specimens subjected to accelerated water immersion.

Figure 7 shows the stress versus strain plots for the large-format 3D-printed WF/aPLA and CF/ABS specimens subjected to accelerated water immersion aging. Different colored curves represent the stress-strain curves for individual specimens. The flexural strength and flexural moduli of WF/aPLA polymer composite specimens were reduced. The strain to failure was also reduced for WF/aPLA specimens, from an average of 2.2% to an average of 1.3%. Similarly, the flexural strength and flexural moduli of CF/ABS polymer composite specimens were reduced. The strain to failure was also reduced for CF/ABS specimens, from 1.6% to 0.94%.

An ANOVA test was used to compare the flexural strength and modulus between the baseline sets and those under moisture absorption. A *p*-value < 0.05 was considered statistically significant. Table 6 shows the comparison of the flexural properties before and after the accelerated water immersion aging test. The results of the ANOVA test show that the changes in both flexural strength and flexural moduli were statistically significant for WF/aPLA and CF/ABS polymer composite specimens.

Significant reductions in mechanical properties have been reported for ABS as well as PLA composites due to the hygrothermal aging process. Jiang et al. [44] reported a significant reduction in the strength of Jute/PLA composites manufactured using conventional methods due to cracking between the fiber and matrix, associated with the differential swelling and weakening of the interface, and also due to the hydrolysis of the matrix, resulting in microcracking.

#### Flexural Test Results of Specimens Subjected to Freeze–Thaw Cycling

Table 7 presents the average flexural strength and flexural modulus of the WF/PLA, WF/aPLA, and CF/ABS sets subjected to freeze–thaw cycling.

**Figure 8:**
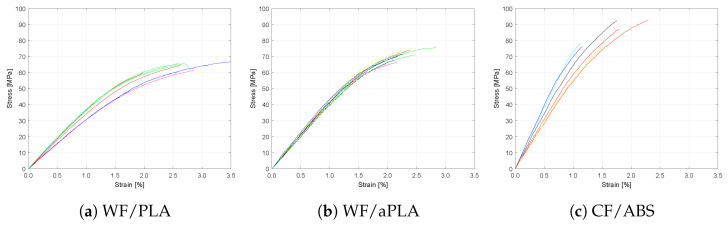
Flexural stress vs. strain curves for specimens subjected to freeze–thaw cycling.

Figure 8 shows the stress versus strain plots for WF/PLA and CF/ABS specimens after freeze–thaw cycling. Different colored curves represent the stress-strain curves for individual specimens. An ANOVA *t*-test, considering a *p*-value < 0.05 as statistically significant, was used to compare the flexural strength and moduli between the baseline sets and those under moisture absorption. Table 8 shows the *p*-values obtained for WF/PLA, WF/aPLA, and CF/ABS.

Freeze–thaw cycling reduced the flexural moduli of WF/PLA and WF/aPLA, with a statistically significant difference from the baseline set of specimens without freeze–thaw conditioning. For WF/PLA, a reduction of 14% in its flexural modulus of elasticity was observed. For WF/aPLA, a reduction of 6% in its flexural modulus of elasticity was observed. No significant change in the flexural properties of CF/ABS specimens was observed due to freeze–thaw cycling.

Freeze–thaw cycling impacted the bio-based materials, affecting the flexural modulus; in the case of CF/ABS, it did not affect its flexural strength and modulus. Similar results were observed for WPC and ABS materials manufactured using conventional manufacturing methods. Darwish [46] studied the effect of freeze–thaw cycling on desktop-scale 3D-printed CF/ABS composites for 300 cycles and found that there was a loss in tensile strength but an increase in the ductility of the material. However, the reductions in strength were small, and the statistical significance of the change was not analyzed. Pilarski et al. [47,48] studied the effect of freeze–thaw cycles on the strength of wood–plastic composites and found that the change in strength was not significant after two full freeze–thaw cycles but was significant after five freeze–thaw cycles. Consistent with these observations, the change in flexural strength was not found to be statistically significant in this study, where only three freeze–thaw cycles were performed. However, the change in the flexural modulus of large-format 3D-printed composites was found to be statistically significant. Turku et al. [49] also reported that freeze-thaw cycles alone did not affect the mechanical properties. The research also concluded that the loss in properties due to water immersion followed by freeze–thaw cycling was similar to the loss in properties solely due to water immersion. Friedrich et al. [50] mentioned that hygrothermal weather cycles reduced the bonding between the fiber and matrix for swelled and frozen fibers. The freeze–thaw cycling protocol followed in this study adopted only 24 h of water immersion at room temperature before freezing. A longer water immersion period at a higher temperature might allow the fibers to absorb water and undergo swelling and freezing and cause debonding between the fiber and matrix, resulting in reduced mechanical properties. Further investigations are necessary to study the effect of the freeze–thaw cycling of large-format 3D-printed thermoplastic wood–plastic composites at higher moisture content.

## 4. Conclusions

The durability of the 3D-printed materials was assessed through water absorption and freeze–thaw cycling accelerated testing. The following conclusions have been drawn from the study.

1. Large-format 3D-printed bio-based polymer composite parts absorb significantly more moisture compared to their synthetic counterparts. The bio-based large-format 3D-printed parts were found to have approximately five times the moisture content of the synthetic material. It is noteworthy that the synthetic material approximately reached moisture content equilibrium, while the bio-based materials did not.
2. The coefficients of moisture expansion for the bio-based materials (WF/PLA and WF/aPLA) were orthotropic, with different coefficients of moisture expansion for the three principal material directions.
3. Bio-based 3D-printed parts are more susceptible to moisture degradation, showing a larger reduction in flexural modulus and flexural strength compared to synthetic material parts.
4. Bio-based 3D-printed materials are more susceptible to degradation due to freeze–thaw cycles, showing a significant reduction in the flexural modulus.

## Figures and Tables

**Table 1:** Moisture content at the end of accelerated moisture exposure.

Set	Number of Specimens	Average Moisture Content (%)	Coefficient of Variation (%)
WF/PLA	10	7.74	4.08
WF/aPLA	6	9.23	0.72
CF/ABS	6	1.35	7.63

**Table 2:** Coefficient of moisture expansion for WF/PLA and WF/aPLA specimens.

Material	$\beta_{1}$ (cv)	$\beta_{2}$ (cv)	$\beta_{3}$ (cv)
WF/PLA	2.13 × 10^−3^ (31%)	2.08 × 10^−3^ (27%)	5.57 × 10^−3^ (7.6%)
WF/aPLA	1.83 × 10^−3^ (10%)	3.26 × 10^−3^ (3.6%)	5.58 × 10^−3^ (6.2%)

**Table 3:** Comparison of coefficients of moisture expansion in principal material directions.

Material Directions Compared	*p*-Value (WF/PLA)	*p*-Value (WF/aPLA)
$\beta_{1}$, $\beta_{2}$, and $\beta_{3}$	1.87 × 10^−14^	9.95 × 10^−11^
$\beta_{1}$ and $\beta_{2}$	0.896	3.25 × 10^−7^
$\beta_{1}$ and $\beta_{3}$	3.80 × 10^−10^	1.51 × 10^−8^
$\beta_{2}$ and $\beta_{3}$	3.36 × 10^−11^	3.88 × 10^−6^

**Table 4:** Baseline flexural properties of WF/PLA, WF/aPLA, and CF/ABS.

Set	Strength (MPa) (Qty/cv)	Modulus (GPa) (Qty/cv)
WF/PLA	64.5 (8/4.3%)	4.2 (8/8.1%)
WF/aPLA	73.2 (10/6.8%)	4.59 (10/4.6%)
CF/ABS	82.5 (10/11%)	6.95 (10/15%)

**Table 5:** Flexural properties of WF/aPLA and CF/ABS after accelerated water immersion aging.

Set	Strength (MPa) (Qty/cv)	Modulus (GPa) (Qty/cv)
WF/aPLA	19.4 (6/4.6%)	2.34 (6/15%)
CF/ABS	42.2 (6/8.9%)	5.74 (6/12%)

**Table 6:** Comparison of flexural properties before and after accelerated water immersion aging.

Set	Baseline	Moisture	% Reduction	*p*-Value
	**Flexural Strength (MPa)**			
WF/aPLA	73.2 (10/6.8%)	19.4 (6/4.7%)	73	1.57 × 10^−13^
CF/ABS	82.5 (10/11%)	42.1 (6/8.9%)	49	4.28 × 10^−8^
	**Flexural Modulus (GPa)**			
WF/aPLA	4.59 (10/4.6%)	2.34 (6/15%)	49	8.57 × 10^−11^
CF/ABS	6.95 (10/15%)	5.74 (6/12%)	18	0.011

**Table 7:** Flexural properties of WF/PLA, WF/aPLA and CF/ABS after freeze–thaw cycling.

Set	Strength (MPa) (Qty/cv)	Modulus (GPa) (Qty/cv)
WF/PLA	64.1 (7/3.8%)	3.64 (7/9.7%)
WF/aPLA	71.9 (10/4.3%)	4.33 (10/4.9%)
CF/ABS	83.9 (6/9.4%)	6.83 (6/16%)

**Table 8:** Comparison of flexural properties before and after freeze–thaw cycling.

Set	Baseline	Freeze–thaw	% Reduction	*p*-Value
	**Flexural Strength (MPa)**			
WF/PLA	64.5 (8/4.3%)	64.1 (7/3.8%)	<1	0.400
WF/aPLA	73.2 (10/6.8%)	71.9 (10/4.3%)	2	0.250
CF/ABS	82.5 (10/11%)	83.9 (6/9.4%)	<1	0.106
	**Flexural Modulus (GPa)**			
WF/PLA	4.21 (8/8.0%)	3.64 (7/9.7%)	14	1.49 × 10^−3^
WF/aPLA	4.59 (10/4.6%)	4.33 (10/4.9%)	6	7.22 × 10^−3^
CF/ABS	6.95 (10/15%)	6.83 (6/16%)	2	3.36 × 10^−3^

## Data Availability

The dataset is available on reasonable request from the authors.

## References

[1] Shah J., Snider B., Clarke T., Kozutsky S., Lacki M., Hosseini A. (2019). Large-scale 3D printers for additive manufacturing: Design considerations and challenges. Int. J. Adv. Manuf. Technol..

[2] Bishop E.G., Leigh S.J. (2020). Using large-scale additive manufacturing as a bridge manufacturing process in response to shortages in personal protective equipment during the COVID-19 outbreak. Int. J. Bioprinting.

[3] Roschli A., Post B.K., Chesser P.C., Sallas M., Love L.J., Gaul K.T. Precast concrete models fabricated with big area additive manufacturing. Proceedings of the 2018 International Solid Freeform Fabrication Symposium.

[4] Bhandari S., Lopez-Anido R., Anderson J. Large scale 3D printed thermoplastic composite forms for precast concrete structures. Proceedings of the Conference & Exhibition on Thermoplastic Composites.

[5] Bhandari S., Lopez-Anido R., Rojas F., LeBihan A. Design and Manufacture of Precast Concrete Formworks Using Polymer Extrusion-Based Large-Scale Additive Manufacturing and Postprocessing. Proceedings of the ASTM International Conference on Additive Manufacturing (ICAM 2021).

[6] Lopez-Anido R., Davids W., Bhandari S., Sheltra C.A., Abdel-Magid B., Erb D.F. Overview of thermoplastic composites in bridge applications. Proceedings of the European Bridge Conference-2022.

[7] Peters B. Additive formwork: 3D printed flexible formwork. Proceedings of the ACADIA.

[8] Bhandari S., Lopez-Anido R.A., Anderson J., Mann A. Large-scale extrusion-based 3D printing for highway culvert rehabilitation. Proceedings of the SPE ANTEC.

[9] Ferrini-Mundy J., Varahramyan K. (2023). 2022 Research Report: R1 Global Impact-Local Relevance.

[10] Liedtka C. (2022). Life Cycle Analysis and Implications of 3D Printed Bio-Based Homes, A Preliminary Study.

[11] Van de Werken N., Tekinalp H., Khanbolouki P., Ozcan S., Williams A., Tehrani M. (2020). Additively manufactured carbon fiber-reinforced composites: State of the art and perspective. Addit. Manuf..

[12] Zhao X., Tekinalp H., Meng X., Ker D., Benson B., Pu Y., Ragauskas A.J., Wang Y., Li K., Webb E. (2019). Poplar as biofiber reinforcement in composites for large-scale 3D printing. ACS Appl. Bio Mater..

[13] Lamm M.E., Wang L., Kishore V., Tekinalp H., Kunc V., Wang J., Gardner D.J., Ozcan S. (2020). Material extrusion additive manufacturing of wood and lignocellulosic filled composites. Polymers.

[14] Talagani M., DorMohammadi S., Dutton R., Godines C., Baid H., Abdi F., Kunc V., Compton B., Simunovic S., Duty C. (2015). Numerical simulation of big area additive manufacturing (3D printing) of a full size car. SAMPE J..

[15] Karbhari V., Chin J., Hunston D., Benmokrane B., Juska T., Morgan R., Lesko J., Sorathia U., Reynaud D. (2003). Durability gap analysis for fiber-reinforced polymer composites in civil infrastructure. J. Compos. Constr..

[16] Gardner D.J., Han Y., Wang L. (2015). Wood–plastic composite technology. Curr. For. Rep..

[17] Yu C.W., Bull J.W. (2006). Durability of Materials and Structures in Building and Civil Engineering.

[18] Nelson W.B. (2009). Accelerated Testing: Statistical Models, Test Plans, and Data Analysis.

[19] Stamboulis A., Baillie C., Garkhail S., Van Melick H., Peijs T. (2000). Environmental durability of flax fibres and their composites based on polypropylene matrix. Appl. Compos. Mater..

[20] Stark N., Gardner D. (2008). Outdoor durability of wood–polymer composites. Wood–Polymer Composites.

[21] Gardner D.J., Bozo A. (2018). Ten-year field study of wood plastic composites in Santiago, Chile: Biological, mechanical and physical property performance. Maderas. Cienc. Tecnol..

[22] Malpot A., Touchard F., Bergamo S. (2017). An investigation of the influence of moisture on fatigue damage mechanisms in a woven glass-fibre-reinforced PA66 composite using acoustic emission and infrared thermography. Compos. Part B Eng..

[23] Pomies F., Carlsson L.A. (1994). Analysis of modulus and strength of dry and wet thermoset and thermoplastic composites loaded in transverse tension. J. Compos. Mater..

[24] Celestine A.D.N., Agrawal V., Runnels B. (2020). Experimental and numerical investigation into mechanical degradation of polymers. Compos. Part B Eng..

[25] Cormier D., Poddar P. (2020). Durability of polymer matrix composites fabricated via additive manufacturing. Durability of Composite Systems.

[26] Banjo A.D., Agrawal V., Auad M.L., Celestine A.D.N. (2022). Moisture-induced changes in the mechanical behavior of 3D printed polymers. Compos. Part C Open Access.

[27] Kakanuru P., Pochiraju K. (2020). Moisture ingress and degradation of additively manufactured PLA, ABS and PLA/SiC composite parts. Addit. Manuf..

[28] Xiao Y., Zhang S., Chen J., Guo B., Chen D. (2023). Mechanical performance of 3D-printed polyethylene fibers and their durability against degradation. Materials.

[29] Dizon J.R.C., Gache C.C.L., Cascolan H.M.S., Cancino L.T., Advincula R.C. (2021). Post-processing of 3D-printed polymers. Technologies.

[30] Afshar A., Mihut D. (2020). Enhancing durability of 3D printed polymer structures by metallization. J. Mater. Sci. Technol..

[31] Głowacki M., Skórczewska K., Lewandowski K., Szewczykowski P., Mazurkiewicz A. (2023). Effect of Shock-Variable Environmental Temperature and Humidity Conditions on 3D-Printed Polymers for Tensile Properties. Polymers.

[32] Grassi G., Spagnolo S.L., Paoletti I. (2019). Fabrication and durability testing of a 3D printed façade for desert climates. Addit. Manuf..

[33] Duty C.E., Kunc V., Compton B., Post B., Erdman D., Smith R., Lind R., Lloyd P., Love L. (2017). Structure and mechanical behavior of Big Area Additive Manufacturing (BAAM) materials. Rapid Prototyp. J..

[34] Somireddy M., Czekanski A. (2020). Anisotropic material behavior of 3D printed composite structures–Material extrusion additive manufacturing. Mater. Des..

[35] Zou R., Xia Y., Liu S., Hu P., Hou W., Hu Q., Shan C. (2016). Isotropic and anisotropic elasticity and yielding of 3D printed material. Compos. Part B Eng..

[36] (2020). Standard Test Method for Moisture Absorption Properties and Equilibrium Conditioning of Polymer Matrix Composite Materials.

[37] Pönninger A., Defoort B. (2003). Determination of the coefficient of moisture expansion (CME). Materials in a Space Environment.

[38] Chaid J.S., Abdallah F.A., Jalil I.M. (2016). Calculation of moisture expansion coefficient of the above knee prosthetic socket lamination materials. J. Eng. Sustain. Dev..

[39] (2015). Standard Practice for Freeze/Thaw Conditioning of Pultruded Fiber Reinforced Polymer (FRP) Composites Used in Structural Designs.

[40] (2007). Standard Test Method for Flexural Properties of Polymer Matrix Composite Materials.

[41] Wang W., Morrell J.J. (2004). Water sorption characteristics of two wood-plastic composites. For. Prod. J..

[42] Kim J.W., Harper D.P., Taylor A.M. (2008). Effect of wood species on water sorption and durability of wood-plastic composites. Wood Fiber Sci..

[43] Huang S.H., Cortes P., Cantwell W. (2006). The influence of moisture on the mechanical properties of wood polymer composites. J. Mater. Sci..

[44] Jiang N., Yu T., Li Y., Pirzada T.J., Marrow T.J. (2019). Hygrothermal aging and structural damage of a jute/poly (lactic acid)(PLA) composite observed by X-ray tomography. Compos. Sci. Technol..

[45] Aniskevich A., Bulderberga O., Stankevics L. (2023). Moisture Sorption and Degradation of Polymer Filaments Used in 3D Printing. Polymers.

[46] Darwish O.M. (2019). Effect of Saline Immersion and Freeze-Thaw Cycles on Performance of Fused Deposition Modelling (FDM) Materials. Ph.D. Thesis.

[47] Pilarski J.M., Matuana L.M. (2006). Durability of wood flour-plastic composites exposed to accelerated freeze–thaw cycling. II. High density polyethylene matrix. J. Appl. Polym. Sci..

[48] Pilarski J.M., Matuana L.M. (2005). Durability of wood flour-plastic composites exposed to accelerated freeze–thaw cycling. Part I. Rigid PVC matrix. J. Vinyl Addit. Technol..

[49] Turku I., Kärki T., Puurtinen A. (2018). Durability of wood plastic composites manufactured from recycled plastic. Heliyon.

[50] Friedrich D., Luible A. (2016). Investigations on ageing of wood-plastic composites for outdoor applications: A meta-analysis using empiric data derived from diverse weathering trials. Constr. Build. Mater..

